# Life-history trade-offs in a generalist digenean from cetaceans: the role of host specificity and environmental factors

**DOI:** 10.1186/s13071-015-1273-8

**Published:** 2015-12-23

**Authors:** Natalia Fraija-Fernández, Mercedes Fernández, Juan A. Raga, Francisco J. Aznar

**Affiliations:** Marine Zoology Unit, Cavanilles Institute of Biodiversity and Evolutionary Biology, Science Park, University of Valencia, PO Box 22085, 46071 Valencia, Spain

**Keywords:** Host specificity, Trade-off, Egg size, Fecundity, Digenea

## Abstract

**Background:**

Adults and larvae of generalist parasites are exposed to diverse hosts and local environmental conditions throughout their life cycles, thus local adaptation is expected to occur through phenotypic plasticity and/or natural selection. We investigated how the combined effect of cryptic host specificity and local selective pressures could shape reproductive traits of a putative generalist parasite in the oceanic realm.

**Methods:**

The LSU rDNA, ITS2 and the mt-COI of individuals of the digenean *Pholeter gastrophilus* (Kossack, 1910) Odhner, 1914 (Heterophyidae Leiper, 1909) from oceanic striped dolphins, *Stenella coeruleoalba* Meyen, and coastal bottlenose dolphins, *Tursiops truncatus* Montagu, in the western Mediterranean were used to elucidate whether worms were conspecific. Infection parameters were compared between both dolphin species. General Linear Mixed Models were used to analyse the influence of host species on four reproductive traits of *P. gastrophilus*: body size, maturity stage (non-gravid/gravid), egg size, and number of eggs *in utero*. AIC values were used to rank competing models, and *p*-values to assess the effect of specific predictors.

**Results:**

Evidence indicated that worms collected from both dolphin species were conspecific. All worms collected were gravid and infection parameters did not differ between dolphin species. However, body size and egg size of individuals of *P. gastrophilus* were significantly larger in striped dolphins. The number of eggs *in utero* did not significantly differ between dolphin species but, for a given body size, worms in bottlenose dolphins harboured more eggs. A trade-off between egg size and egg number was found in worms from both dolphin species, with a higher slope in striped dolphins.

**Conclusions:**

Apparently, striped dolphin is a more suitable host for *P. gastrophilus*, but reproductive investment seems to be adapted to the habitat where the life-cycle develops. Worms from striped dolphins likely face the problem of finding intermediate hosts in the oceanic realm and apparently invest into offspring size to enhance the early survival of larvae and the potential to multiply asexually within the first intermediate host. The small-sized worms from bottlenose dolphins would be adapted to reproduce early because of higher adult mortality, generating smaller and numerous eggs in a coastal habitat where chances of transmission are presumably higher.

## Background

Host specificity is a measure of the degree to which parasites species can exploit different host species. Differences of specificity are a matter of degree rather than kind: at one extreme, specialist parasites can exploit only one or very few host species; at the other extreme, generalist parasites can infect and reproduce in a number of host species [1]. According to the encounter/compatibility paradigm, the degree of specificity of any parasite is determined by the action of two sequential filters. The ‘encounter’ filter prevents infections of potential hosts that cannot contact the parasite, whereas the ‘compatibility’ filter excludes contacted hosts in which the parasite cannot find the appropriate resources and/or escape or deter the host’s defenses [2, 3]. If the contacted hosts are suitable but suboptimal, the compatibility filter can still negatively affect fitness components of the parasite.

A fundamental question regarding host specificity is the extent to which a putative generalist parasite performs equally well in all exploited host species, namely, whether these hosts are all equally compatible for the parasite [4]. This question has important implications, not only for understanding the evolution of specificity, but also for population dynamics and epidemiology of generalist parasites [5]. In some cases, there is little evidence that sympatric hosts impact differently the performance of a putative generalist parasite, e.g. [5]. Other studies, however, have reported on significant host species effects on fitness-related traits of the parasite such as dwarfism and impaired reproduction in presumably suboptimal hosts, e.g. [6–8]. Other studies have discovered more subtle effects on parasite’s life history traits (e.g. fecundity) of host species that, at first glance, seemed to be equally suitable for the parasite, e.g., [9–11]. It should be stressed, however, that some putative generalist parasites have later been re-interpreted, based on molecular evidence, as cryptic species complexes, each species being adapted to a different host [12, 13].

Differences of performance of a generalist parasite among host species usually result from the diverse conditions each host species provides [14, 15]. For instance, a parasite can be adapted to some host species, but is also able to reproduce, with reduced success, in related hosts because, in the latter, the parasite suffers a shortage of trophic resources or harsh physiological conditions, or incur higher costs in the face of host immune responses [16, 17]. However, individual life history traits are constrained, not only by phylogenetic, physical or developmental factors, but also by trade-offs with other traits [18]. Selection pressures operate on the whole life-cycle and, therefore, trade-offs are optimised for the specific environment where the life-cycle develops [19–21]. Therefore, it is important to consider not only the microhabitat conditions each host species provides, but also where each host lives. To our knowledge, few studies have analysed how the combined effect of host species constraints, and local selective pressures on the life-cycle, could shape life history traits of a generalist parasite, e.g. [5].

In this paper, we investigate this issue in a digenean infecting two cetacean species that occur in different habitats, i.e. coastal and oceanic. *Pholeter gastrophilus* (Kossack, 1910) Odhner, 1914 is a member of the family Heterophyidae Leiper, 1909 that has been reported as adult in at least 17 odontocete species inhabiting coastal, oceanic and even freshwater habitats worldwide [22]. Worms live encysted inside fibrotic nodules in the wall of glandular chambers of the stomach [23]. Eggs are released to the stomach lumen through a narrow duct that stems from the cyst [24, 25]. The life-cycle of *P. gastrophilus* is not known but, based on the broad ecological distribution of its definitive hosts and evidence on the life-cycles of other heterophyids, it can be postulated that molluscs act as first intermediate hosts, and a wide array of invertebrates and/or fish are second intermediate hosts [23, 25].

In the western Mediterranean, *P. gastrophilus* has been reported in four sympatric cetacean species, i.e. the Risso’s dolphin, *Grampus griseus* G. Cuvier, the long-finned pilot whale, *Globicephala melas* Traill and, especially, the bottlenose dolphin, *Tursiops truncatus* Montagu, and the striped dolphin, *Stenella coeruleoalba* Meyen [26–30]. Gravid specimens of *P. gastrophilus* have been found in all these host species, but a more rigorous analysis, of the extent to which host species may affect growth and reproduction of the parasite, has never been carried out. Interestingly, the Mediterranean hosts of *P. gastrophilus* live in ecologically distinct habitats. In particular, striped dolphins, Risso’s dolphin and long-finned pilot whales are primarily oceanic species, whereas bottlenose dolphins favour more coastal waters [31, 32]. The extent to which environmental factors associated to oceanic vs. coastal realm also influence the reproductive strategies of *P. gastrophilus* is an open, interesting question.

In this study, we examined patterns of host specificity of *P. gastrophilus* collected from bottlenose and striped dolphins in the western Mediterranean with four specific aims. First, we ascertained if worms collected from both host species belong to a single generalist taxon or represent a species complex. Second, we compared infection parameters of *P. gastrophilus* between both dolphin species. Third, we analysed host-parasite compatibility based on a comparison of four reproduction-related traits, i.e. body size, presence/absence of eggs, number of eggs *in utero*, and egg size. Finally, we explored whether a phenotypic trade-off between egg number and egg size occurred [33–35], and whether it was optimised differently for each host species. We acknowledge from the outset that the amount of data that can be gathered from this system is limited. However, the results obtained strongly suggest how host suitability and environmental conditions can modulate some key features of the life history strategy of an oceanic parasite.

### Ethical approval

Permission and funding to collect stranded dolphins was given by the Wildlife Service of the Valencian Regional Government, Spain, which is the official institution in charge of managing and protecting wildlife in the region. No ethics board was involved because animals were collected after their natural death.

## Methods

### Sample collection

A total of 39 striped dolphins and 21 bottlenose dolphins stranded on the Mediterranean coast of Spain (40°25′N, 0°26′E and 37°58′N, 0°41′W) were collected during 1990–2005. Only well-preserved carcasses (state 1–3 *sensu* [36]) were selected for analysis. Animals were brought to the laboratory and immediately necropsied; the stomach was removed and frozen at -20 °C for later examination. After thawing, each stomach chamber was examined separately for the presence of *P. gastrophilus*. Nodules were detected through visual and tactile screening and incisions were made on each nodule to confirm the presence of *P. gastrophilus*. When positive, the nodule was removed and carefully cut into slices to collect and count all worms. Finally, the stomach content was filtered over a sieve with 0.02 mm mesh spacing to collect worms that were free in the lumen. Specimens of *P. gastrophilus* were washed in 0.9 % saline and fixed in absolute ethanol for molecular analysis or 70 % ethanol for morphometric analysis.

### Molecular analyses

Single individuals of *P. gastrophilus* collected from five bottlenose dolphins, and six worms collected from five striped dolphins were used for molecular analysis to elucidate whether all worms were conspecific. Genomic DNA was extracted from individual worms using a standard phenol-chloroform protocol. Partial large subunit (LSU) rDNA was amplified in two specimens of *P. gastrophilus* from each dolphin species using primers LSU5 [37] and LSU1500R [38]. The ITS2 rDNA was amplified in four worms from each dolphin species using primers 3S [39] and ITS2.2 [40]. The mitochondrial COI was amplified from five and three worms from striped and bottlenose dolphins, respectively, using primers JB3 [41] and JB4.5 [42]. An additional primer, 300 F [43] was used as an internal primer for sequencing the LSU rDNA. Thermocycling profiles for gene amplification were as follows: for the LSU rDNA, initial denaturation at 94 °C for 3 min, 40 cycles of 94 °C for 30 s, 56 °C for 30 s, 72 °C for 2 min, and a final extension at 72 °C for 7 min [38]; for the ITS2 rDNA, initial denaturation at 95 °C for 3 min, 40 cycles of 94 °C for 50 s, 56 °C for 50 s, 72 °C for 80 s, and a final extension at 72 °C for 4 min [13]; and for the mitochondrial COI gene, initial denaturation at 94 °C for 5 min, 40 cycles of 92 °C for 30 s, 45.6 °C for 45 s, 72° for 90 s, and a final extension at 72 °C for 10 min [42]. Amplicons were purified with a GFX PCR DNA and Gel Band Purifying Kit (GE Healthcare Life Sciences, Buckinghamshire, UK) and cycle sequenced on an Applied Biosystems 3730 DNA Analyser, using Big Dye version 1.1. Contiguous sequences were assembled and analysed using BioEdit v.7.0.5.3 [44]. Sequences are available online in the GenBank with accession numbers as follows: sequences for *P. gastrophilus* from striped dolphins [KT883852 (LSU rDNA); KT883854 (ITS2 rDNA); KT883856 (mt COI)] and sequences of *P. gastrophilus* from bottlenose dolphins [KT883853 (LSU rDNA); KT883855 (ITS2 rDNA); KT883857 (mt COI).

### Comparison of infection parameters

The 95 % confidence intervals (CI) for prevalence of *P. gastrophilus* in striped and bottlenose dolphins was calculated with Sterne’s exact method [45], and for mean values of intensity, with the bias-corrected and accelerated bootstrap method using 20,000 replications [46]. Prevalence was compared between both dolphin species with Fisher’s exact test and mean intensity with bootstrap t-tests [46]. Also, a Brunner-Munzel test was used to compare the probability that the intensity of *P. gastrophilus* in individual hosts from one dolphin species was higher than that in individual hosts from the other dolphin species [47]. These analyses were carried out with the free software Quantitative Parasitology v. 3 [46].

### Comparison of life-history traits

**Fig. 1:**
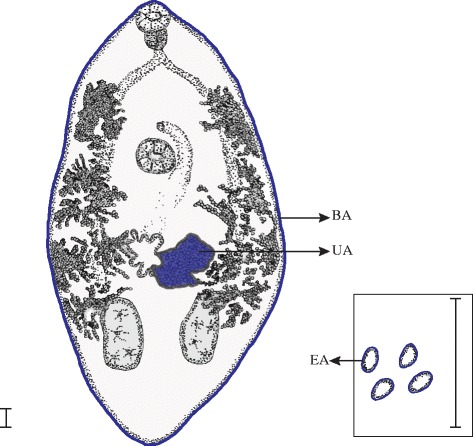
Schematic drawing of a specimen of *Pholeter gastrophilus* and its eggs (inset)*.* Colour lines represent measurements taken for the study. *Abbreviations*: BA, body area; UA, uterine area covered by eggs; EA, egg area. *Scale-bars*: 0.1 mm

Individuals of *P. gastrophilus* collected from five freshly dead striped dolphins (*n* = 140 worms) and five bottlenose dolphins (*n* = 97) were used for comparison of four reproductive traits: body size, maturity stage (gravid/non-gravid), egg size, and number of eggs *in utero*. Parasites were stained with iron acetocarmine; excess of carmine was removed with HCl in 70 % ethanol. Specimens were dehydrated through a graded ethanol series, cleared with dimethyl phthalate and mounted as permanent preparations in Canada balsam. Body area and uterine area filled with eggs were drawn for each individual using a stereomicroscope (×40) connected to a drawing tube. In platyhelminths, areas provide a good proxy of body size and the size of irregular elements [8, 48, 49]. Empty portions of the uterus could not be observed because the uterine wall is very faint and frequently obscured by vitelline follicles. The area of 10 randomly selected eggs were also drawn using a light microscope (×200) connected to a drawing tube (Fig. 1). A single value of egg area per worm was obtained by averaging measurements from 10 eggs. Egg areas were preferred over egg volumes (calculated assuming regular shapes) for consistency with the other measurements. All areas were calculated with the program Image Tool v.3.0 [50]. The number of eggs *in utero* was calculated by dividing the uterine area filled with eggs by the average egg area for each parasite. We confirmed that this method was a good proxy for the number of eggs *in utero* as follows. After calculating the number of eggs *in utero* as described above, we de-mounted 10 randomly chosen worms from striped dolphins (2 per individual host) and 10 from bottlenose dolphins (2 per individual host). Each worm was torn apart in 2000 μl of saline to release all eggs. The solution was homogenised with a magnetic stirrer and eggs from 3 samples of 20 μl were counted with the aid of a Bürker chamber following the manufacturer’s recommendations (OptikLabor, Lancing, UK). The average of the three counts was used as a measure of the number of eggs per μl. The relationship between the number of eggs *in utero* calculated with the two methods was linear, fairly strong and highly significant (Pearson’s correlation, *r* = 0.67, *p* = 0.001).

General Linear Mixed Models (GLMMs) with type III sum of squares were used to analyse the influence of different predictors on body area, number of eggs *in utero* and egg area of *P. gastrophilus* [51]. Morphometric variables were log10-transformed prior to analysis. We used the values of Akaike Information Criteria (AIC) to rank competing models with different numbers of fixed and random parameters. The model with minimum AIC was considered the “best model”, and the rest of the models were ranked according to differences in their AIC values [52]. Models with values of ΔAIC ≤ 2 were considered to have substantial empirical support, whereas those having ΔAIC > 4 were assumed to have much less support [53]. It was also assumed that models with Akaike weights (*w*_*i*_) ≤ 0.01 were unlikely to be the “true” model [53] and, therefore, they are not shown in the tables. Fixed parameters in all candidate models, excluding the intercept, were also tested for statistical significance using F- or t-tests.

In models accounting for variability in worm body area, ‘host species’ (factor),‘intensity’ (covariate), and ‘host species*intensity’ were considered as potential predictors, i.e. fixed factors. ‘Intensity’ was included to investigate possible crowding effects on body size [49]. ‘Individual host’ was considered as a potential random parameter, i.e. a random intercepts model. In models accounting for both variability in number of eggs *in utero* and egg area, ‘host species’, ‘worm body area’ (covariate), and ‘host species*worm body area’ were considered as potential predictors. ‘Individual host’ and ‘worm body area’ were considered as potential random parameters. In this case, the random part of the models were allowed to include ‘individual host’ (i.e. a random intercepts model), ‘individual host‘ + ’worm body area’ (i.e. a random intercepts and random slopes model), and ‘individual host‘ + ’worm body area’ + covariance between intercepts and slopes (i.e. an unstructured model; see [51]).

The trade-off between egg size and egg number was also assessed through GLMMs. In this case we used Type I sum of squares since we wanted to control for the effect of parasite body area on either number of eggs *in utero* and egg area [54]. ‘Worm body area’ (covariate), ‘egg area’ (covariate), ‘host species’, ‘host species*worm body area’ and ‘host species*egg area’ were entered sequentially in the models as potential predictors. ‘Individual host’, ‘worm body area’ and ‘egg area’ were considered as potential random parameters. In this case, the random part of the models were allowed to include (i) ‘individual host’; (ii) ‘individual host‘ + ’worm body area’; (iii) ‘individual host‘ + ’egg area’; (iv) ‘individual host‘ + ’worm body area‘ + ’egg area’; and (v) ‘individual host‘ + ’worm body area‘ + ’egg area‘ + covariance between intercepts and slopes. GLMMs were implemented with SPSS for Macintosh, v. 19.0.

## Results

### Molecular analyses

We obtained four partial LSU rDNA sequences for *P. gastrophilus* (816-1,285 bp long). The eight sequences from each, the ribosomal ITS2 spacer and the mitochondrial COI gene varied between 466 and 515 bp, and between 396 and 446 bp, respectively. Comparison of pairwise divergence for each gene showed that the aligned portions of the sequences of all specimens of *P. gastrophilus* were identical.

### Infection parameters

The prevalences (95 % CI) of *P. gastrophilus* in striped and bottlenose dolphins were 56.4 % (40.5–71.3) and 57.1 % (35.4–76.7), respectively; the difference was not significant (Fisher’s test, *p* = 1). Mean intensity of *P. gastrophilus* also did not differ between both host species: 77.7 (50.7–134.6) worms per infected host in striped dolphins vs. 249.2 (82.9–644.4) worms per infected host in bottlenose dolphins (t = -1.321, *p* = 0.277). In addition, the Brunner-Munzel test was not significant (*p* = 0.847).

### Comparison of life-history traits

All individuals of *P. gastrophilus* collected from both, striped dolphins and bottlenose dolphins, were gravid. Mean values of worm body area, number of eggs *in utero* and egg area are shown in Table 1. The best GLMM for worm body area included only ‘host species’ and ‘host individual’ effects; any other model received substantially less empirical support (Table 2). In the subset of models with *w*_*i*_ > 0.01, ‘host species’ but not ‘intensity’, was found to be a highly significant predictor of worm body area (Table 2). The average body area of *P. gastrophilus* in striped dolphins was nearly twice that found in bottlenose dolphins, and the difference was consistent regardless of host individual (Table 1).

**Table 1:** Mean values (± standard deviation, SD) and coefficient of variation (CV in %) of body area, egg area and number of eggs *in utero* of individuals of *Pholeter gastrophilus* collected from five striped dolphins, *Stenella coeruleoalba*, and five bottlenose dolphins, *Tursiops truncatus*, stranded along the Mediterranean coast of Spain

Host	Intensity	Body area (mm^2^)	CV	Egg area (μm^2^)	CV	Egg number	CV	Uterus area (mm^2^)	CV
*Stenella coeruleoalba*									
Host 1	34	3.01 ± 0.77	25.6	227 ± 46	20.3	4,170 ± 2,391	57.3	0.94 ± 0.53	56.8
Host 2	27	2.51 ± 0.42	15.5	224 ± 44	19.5	2,148 ± 2,183	101.6	0.43 ± 0.40	92.8
Host 3	35	3.63 ± 0.86	23.6	220 ± 39	17.8	5,076 ± 3,414	67.3	1.07 ± 0.68	63.5
Host 4	24	4.09 ± 0.77	18.8	204 ± 30	14.6	6,814 ± 2,945	43.2	1.36 ± 0.57	42.2
Host 5	20	4.31 ± 0.86	20.0	237 ± 36	15.0	5,984 ± 1,556	26.0	1.43 ± 0.44	31.0
Total	140	3.44 ± 0.98	28.4	222 ± 41	18.3	4,719 ± 3,040	64.4	1.02 ± 0.64	62.6
Average per host	28	3.5 ± 0.75	21.43	222 ± 12	5.4	4,838 ± 1,799	37.2	1.05 ± 0.40	38.1
*Tursiops truncatus*									
Host 1	33	2.22 ± 0.96	43.2	233 ± 39	16.9	3,914 ± 3,068	78.4	0.88 ± 0.68	77.4
Host 2	5	0.92 ± 0.33	36.1	185 ± 20	10.6	2,125 ± 879	41.4	0.40 ± 0.18	44.7
Host 3	18	1.47 ± 0.23	15.6	149 ± 15	10.4	3,209 ± 1,741	54.2	0.48 ± 0.26	53.0
Host 4	19	1.27 ± 0.28	22.0	154 ± 17	11.2	1,480 ± 1,370	92.2	0.23 ± 0.23	96.5
Host 5	22	2.51 ± 0.53	21.3	169 ± 26	15.3	8,100 ± 2,168	26.8	1.37 ± 0.40	29.2
Total	97	1.89 ± 0.82	43.5	185 ± 45	24.4	4,164 ± 3,236	77.7	0.77 ± 0.61	80.2
Average per host	19.4	1.68 ± 0.66	39.3	178 ± 34	19.1	3,765 ± 2,599	69.0	0.67 ± 0.46	68.7

**Table 2:** General Linear Mixed Models with type III sum of squares accounting for the effect of host species (factor) and trematode intensity (covariate) on body area of individuals of *Pholeter gastrophilus* collected from striped and bottlenose dolphins

Model			ΔAIC	*w*_*i*_	Predictor	t	df	*p*
Fixed effects	Random effects	Covariance structure						
Intercept + HS	HI	RI	0.00	0.922	HS	3.85	7.61	**0.005**
Intercept	HI	RI	6.39	0.038	-	-		-
Intercept + HS + I	HI	RI	6.63	0.034	HS	2.84	6.80	**0.026**
					I	1.38	7.31	0.209

Models are arranged by increase of Akaike information criterion (AIC) and decrease of Akaike weight (*w*_*i*_). Models with *w*_*i*_ < 0.01 are not shown. The probability associated to each fixed effect is also given; significant values are in bold. *Abbreviations*: *HS* host species, *HI* host individual, *RI* random intercept, *I* intensity

**Fig. 2:**
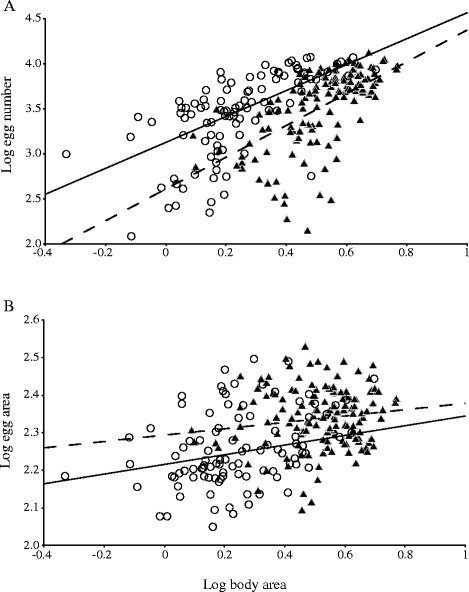
Scatterplots showing the relationship of life-history traits in *Pholeter gastrophilus*. Scatterplots showing the relationship between log_10_-transformed values of (**a**) body area and number of eggs *in utero* and (**b**) body area and egg area of 237 individuals of the digenean *Pholeter gastrophilus* in five striped dolphins, *Stenella coeruleoalba* (triangles; *n* = 140) and five bottlenose dolphins, *Tursiops truncatus* (circles; *n* = 97) from the western Mediterranean. Regression lines were obtained from the best model that fit the data for striped dolphins (dashed line) and bottlenose dolphins (solid line)

The best GLMM for the number of eggs *in utero* included ‘host species’, ‘worm body area’ and the interaction between both variables as fixed predictors, and ‘host individual’ as a random factor (Table 3). However, three competing models received also substantial support (∆AIC < 4; *w*_*i*_ > 0.10), and all included both ‘host species’ and ‘worm body area’ as fixed factors (Table 3). These two factors also were significant predictors in all models (*p* ≤ 0.015), but their interaction was not (Table 3). These results indicate that larger worms harboured more eggs regardless of host species and, for a given body size, the number of eggs *in utero* was significantly higher in worms from bottlenose dolphins (Fig. 2a). Note, however, that in all the models excluding ‘worm body area’ (all with ∆AIC > 4), the effect of ‘host species’ was not significant (*p* > 0.05). In other words, absolute fecundity was similar between worms from both host species (Fig. 2a).

**Table 3:** General Linear Mixed Models with type III sum of squares accounting for the effect of host species (factor) and worm body area (covariate) on the number of eggs *in utero* of *Pholeter gastrophilus* collected from striped and bottlenose dolphins

Model			ΔAIC	*w*_*i*_	Predictor	t	df	*p*
Fixed effects	Random effects	Covariance structure						
Intercept + HS + BA + HS*BA	HI	RI	0.00	0.355	HS	-2.58	32.68	**0.015**
					BA	6.24	190.29	**<0.005**
					HS*BA	0.64	190.29	0.526
Intercept + HS + BA	HI	RI	0.07	0.342	HS	-2.97	9.82	**0.014**
					BA	9.32	189.55	**<0.005**
Intercept + HS + BA + HS*BA	HI + BA	RI + RS	2.00	0.130	HS	-2.58	32.68	**0.015**
					BA	6.24	190.29	**<0.005**
					HS*BA	0.64	190.29	0.526
Intercept + HS + BA	HI + BA	RI + RS	2.07	0.126	HS	-2.97	9.82	**0.014**
					BA	9.32	189.55	**<0.005**
Intercept + BA	HI	RI	4.67	0.034	BA	8.70	149.45	**<0.005**

Models are arranged by increase of Akaike information criterion (AIC) and decrease of Akaike weight (*w*_*i*_). Models with *w*_*i*_ < 0.01 are not shown. The probability associated to each fixed effect is also given; significant values are in bold. *Abbreviations*: *HS* host species, *BA* worm body area, *HI* host individual, *RI* random intercept, *RS* random slope

The best GLMM for egg size included only ‘body area’ as a fixed, highly significant predictor, and ‘host individual’ as a random factor (Table 4). Two additional models also received substantial empirical support (∆AIC < 4; *w*_*i*_ > 0.10), and one of them also included ‘host species’ as an additional predictor (Table 4). The effect of ‘body area’, but not ‘host species’, was statistically significant (Table 4). When ‘body area’ was removed from models, empirical support decreased substantially (∆AIC ≥ 6) and the effect of ‘host species’ was statistically significant (t = 2.74, df = 8.28, *p* = 0.025). Accordingly, the size of the eggs in *P. gastrophilus* increased with worm body size, and the difference of egg size between host species resulted from corresponding differences in worm body size, being larger in striped dolphins (Fig. 2b). This pattern was consistent across host individuals, except in the case of one individual bottlenose dolphin that harboured worms with similar values as those observed in striped dolphins (Table 1).

**Table 4:** General Linear Mixed Models with type III sum of squares accounting for the effect of host species (factor) and worm body area (covariate) on egg area of individuals of the digenean *Pholeter gastrophilus* collected from striped and bottlenose dolphins

Model			ΔAIC	*w*_*i*_	Predictor	t	df	*p*
Fixed effects	Random effects	Covariance structure						
Intercept + BA	HI	RI	0	0.506	BA	3.66	165.54	**<0.005**
Intercept + BA	HI + BA	RI + RS	2.00	0.186	BA	3.66	165.53	**<0.005**
Intercept + HS + BA	HI	RI	2.33	0.158	HS	1.58	10.05	0.145
					BA	2.98	223.38	**<0.005**
Intercept + HS + BA	HI + BA	RI + RS	4.33	0.058	HS	1.58	10.04	0.145
					BA	2.98	223.35	**<0.005**
Intercept + HS + BA + HS*BA	HI	RI	5.51	0.032	HS	1.40	23.44	0.175
					BA	2.27	222.45	**0.024**
					HS*BA	-0.23	222.45	0.820
Intercept + HS	HI	RI	6.45	0.020	HS	2.74	8.28	**0.025**

Models are arranged by increase of Akaike information criterion (AIC) and decrease of Akaike weight (*w*_*i*_). Models with *w*_*i*_ < 0.01 are not shown. The probability associated to each fixed effect is also given; significant values are in bold. *Abbreviations*: *BA* worm body area, *HI* host individual, *RI* random intercept, *RS* random slope, *HS* host species

**Fig. 3:**
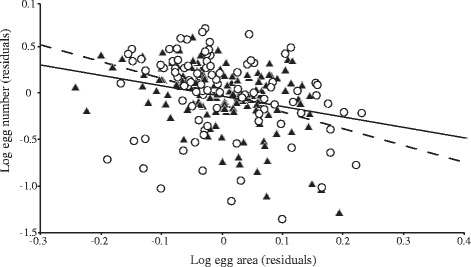
Scatterplot showing the relationship between body-size corrected residuals of egg area and number of eggs *in utero* of the digenean *Pholeter gastrophilus* in striped dolphins, *Stenella coeruleoalba* (triangles) and bottlenose dolphins, *Tursiops truncatus* (circles) from the western Mediterranean. Regression lines were obtained from the best model that fit the data for striped dolphins (dashed line) and bottlenose dolphins (solid line)

When evaluating a trade-off between number of eggs *in utero* and egg area, five models received substantial empirical support (Δ AIC ≤ 2*w*_*i*_ > 0.085) (Table 5). ‘Worm body area’ and ‘egg area’ were included in all models and also received strong statistically significant support (*p* ≤ 0.005). After controlling for body area, a negative relationship was found between the egg number and egg area in all five models (Fig. 3). Parameter for ‘egg area’ in the best model (SE) was -0.88 (0.47); in the other four models values ranged from -0.95 (0.46) to -0.82 (0.48). ‘Host species’ and ‘host species*egg area’ were also selected in all five best supported models; apparently, the negative relationship between ‘number of eggs’ and ‘egg area’ was steeper in worms from striped dolphins (Fig. 3). However, ‘host species’, but not ‘host species*egg area’, was statistically significant (Table 5).

**Table 5:** General Linear Mixed Models with type I sum of squares accounting for the trade-off between egg number and egg area of individuals of *Pholeter gastrophilus* collected from striped and bottlenose dolphins, controlling for worm body area (covariate)

Model			ΔAIC	*w*_*i*_	Predictor	F	df	*p*
Fixed effects	Random effects	Covariance structure						
Intercept + BA + EA + HS + HS*BA + HS*EA	HI	RI	0	0.231	BA	24.93	1, 9.6	**<0.005**
					EA	31.18	1, 120.4	**<0.005**
					HS	5.66	1, 9.7	**0.040**
					HS*BA	0.27	1, 195.8	0.601
					HS*EA	2.45	1, 198.2	0.119
Intercept + BA + EA + HS + HS*EA	HI	RI	0.36	0.193	BA	24.49	1, 9.3	**<0.005**
					EA	31.03	1, 119.2	**<0.005**
					HS	5.85	1, 9.5	**0.037**
					HS*EA	1.99	1, 199.0	0.160
Intercept + BA + EA + HS + HS*BA + HS*EA	HI + EA	RI + RS	1.66	0.101	BA	24.42	1, 9.4	**<0.005**
					EA	29.53	1, 114.1	**<0.005**
					HS	5.51	1, 9.5	**0.042**
					HS*BA	0.19	1, 196.0	0.661
					HS*EA	2.67	1, 192.1	0.104
Intercept + BA + EA + HS + HS*EA	HI + EA	RI + RS	1.92	0.089	BA	24.09	1, 9.2	**<0.005**
					EA	29.35	1, 113.0	**<0.005**
					HS	5.70	1, 9.4	**0.040**
					HS*EA	2.24	1, 191.4	0.136
Intercept + BA + EA + HS + HS*BA + HS*EA	HI + BA	RI + RS	2.00	0.085	BA	24.93	1, 9.6	**<0.005**
					EA	31.18	1, 120.4	**<0.005**
					HS	5.66	1, 9.7	**0.040**
					HS*BA	0.27	1, 195.8	0.601
					HS*EA	2.45	1, 198.2	0.119
Intercept + BA + EA + HS + HS*EA	HI + BA	RI + RS	2.36	0.071	BA	24.49	1, 9.3	**<0.005**
					EA	31.03	1, 119.2	**<0.005**
					HS	5.85	1, 9.5	**0.037**
					HS*EA	1.99	1, 199.0	0.160
Intercept + BA + EA + HS	HI	RI	3.00	0.052	BA	22.76	1, 9.1	**<0.005**
					EA	39.23	1, 137.1	**<0.005**
					HS	4.89	1, 9.2	0.054
Intercept + BA + EA + HS + HS*BA	HI	RI	3.14	0.048	BA	22.74	1, 9.2	**<0.005**
					EA	39.41	1, 137.9	**<0.005**
					HS	4.65	1, 9.3	0.058
					HS*BA	0.30	1, 201.1	0.583
Intercept + BA + EA + HS + HS*BA + HS*EA	HI + BA + EA	RI + RS	4.65	0.023	BA	27.79	1, 3133.1	**<0.005**
					EA	33.21	1, 352.7	**<0.005**
					HS	6.29	1, 3119.3	**0.012**
					HS*BA	0.27	1, 323.9	0.605
					HS*EA	2.66	1, 298.4	0.104
Intercept + BA + EA + HS	HI + EA	RI + RS	4.84	0.021	BA	23.29	1, 9.0	**<0.005**
					EA	36.91	1, 91.0	**<0.005**
					HS	4.36	1, 9.2	0.066

Models are arranged by increase of Akaike information criterion (AIC) and decrease of Akaike weight (*w*_*i*_). Models with *w*_*i*_ < 0.01 are not shown. The probability associated to each fixed effect is also given; significant values are in bold. *Abbreviations*: *BA* worm body area, *EA* egg area, *HS* host species, *HI* host individual, *RI* random intercept, *RS* random slope

## Discussion

*Pholeter gastrophilus* is considered a generalist species with a large range of hosts and a worldwide distribution. This raises the question as to whether this parasite might actually represent a complex of cryptic species [23], a phenomenon that has been observed in other digeneans, e.g. [55]. The lack of variation in the sequences of the LSU and ITS2 rDNA and mtCOI, respectively, suggests that individuals of *P. gastrophilus* collected from the striped and bottlenose dolphins are conspecific. Ribosomal genes exhibit some variable sites with phylogenetic information for congeneric taxa [13] whereas mitochondrial genes usually accumulate much higher nucleotide substitutions than rDNA, thus being especially useful for discriminating closely related species [56, 57]. The absence of genetic differences in *P. gastrophilus* associated with host species could be related to the fact that striped and bottlenose dolphins utilise partially overlapping habitats [32] and share some preys in the study area [58, 59], thus enabling gene flow in the parasite population. Nevertheless, significant population structure might still occur in the neritic vs. oceanic populations of *P. gastrophilus* (see below). To detect intraspecific structure at this spatial scale, sequences of a much larger number of worms, and use of additional genetic markers are required (see, e.g. [60]).

Infection levels of *P. gastrophilus* were similar in both striped and bottlenose dolphins; prevalence was virtually identical, and differences in worm intensity were not significant. To understand how the contact and/or compatibility filters generate this similarity, we would need information about (i) the diet of each dolphin species; (ii) the life cycle of *P. gastrophilus*; and (iii) the potential effect of hosts’ physiological or immunological factors on mortality rates of parasites. The latter factor cannot be ascertained unless experimental infections are carried out. Concerning host-parasite contact, in the study area, striped dolphins feed primarily on oceanic mesopelagic fish and cephalopods [58], although they may consume some neritic fish, e.g. juvenile hake, *Merluccius merluccius*, or cephalopods, e.g. *Illex coindetii* [61]. In contrast, bottlenose dolphins feed largely on demersal neritic fish (especially hake) and, to a lesser extent, on benthic cephalopods [59]. Thus, it is puzzling how *P. gastrophilus* is able to infect a sizeable proportion of two dolphin species with such a small overlap in habitat and diet. The problem is compounded because, in the study area, this parasite also infects other oceanic cetaceans that feed almost exclusively on mesopelagic cephalopods [28, 29]. Unfortunately, parasitological surveys have failed to find infective stages of *P. gastrophilus* in the main prey of striped and bottlenose dolphins, see [62, 63]. Based on data from other digeneans infecting pelagic vertebrates [64–66] we can postulate that *P. gastrophilus* extensively exploits the food web to reach its definitive hosts, but it is unclear to what extent the transmission routes are different depending on each cetacean species.

In parasitic platyhelminths, a large body size has been linked to a suite of life history traits including low mortality, long maturation time, slow growth rate and high reproductive output [35]. In *P. gastrophilus*, a larger body size was correlated with both a higher number of eggs *in utero* and larger eggs, similarly as in other digeneans, e.g. [67, 68]. Also, individuals of *P. gastrophilus* were able to mature and reproduce in both dolphin hosts, but worms were significantly larger in striped dolphins and also harboured larger eggs. These differences were consistent regardless of substantial variability associated to host individual, and did not appear to be confounded by differential density-dependence [5]. In fact, bottlenose dolphins provided a larger microhabitat (stomach area: 520 cm^2^ vs. 297 cm^2^ in striped dolphins, F.J. Aznar, unpub. obs.) but harboured smaller parasites that produced smaller eggs.

The larger investment in body size and egg size of *P. gastrophilus* in striped dolphins tentatively suggests that they are more suitable hosts than bottlenose dolphins. The absolute number of eggs *in utero* did not differ between hosts, but this measure of fecundity is just a ‘snapshot’ of the total egg output that a digenean can produce throughout its lifetime [69]. In digeneans, the adult size has a significant positive effect on the total reproductive output [35], thus we cannot rule out that worms from striped dolphins also have a higher overall fecundity. On the other hand, if bottlenose dolphins impose higher adult mortality rates of *P. gastrophilus*, shorter parasite maturation times and smaller body sizes should be favoured by natural selection (see [34]). This would explain why, for a given body size, worms from bottlenose dolphins had a relatively higher number of eggs *in utero*. Although the reasons for such contrasting host suitability are unknown, the possibility that differences in host’s immune response play a role should not be underestimated, since infection with *P. gastrophilus* elicit a significant immune reaction associated to the formation of fibrotic nodules [70].

According to the above discussion, individuals of *P. gastrophilus* seem to adopt different reproductive strategies depending on the host species, i.e. larger worms in the seemingly suitable hosts (striped dolphins) allocate resources for both somatic growth and production of large eggs, whereas smaller worms in the presumably suboptimal hosts (bottlenose dolphins) primarily allocate resources for egg production. The reasons why the absolute number of eggs *in utero* was not significantly higher in worms from striped dolphins deserve further attention. Perhaps there are spatial constraints on the amount of eggs that the uterus can harbour [71], so the larger body size (= uterus) in worms from striped dolphins would be inconsequential for harbouring more eggs if eggs are also larger. A second, non-exclusive hypothesis is that a trade-off could exist between egg size and egg number so that worms in striped dolphins produce larger eggs at the expense of lowering fecundity (see [72, 73]). In fact, we obtained evidence of such trade-off, not only in striped dolphins, but also in bottlenose dolphins. This would empirically confirm that parasites could not equally invest into both quantity and quality of offspring [74, 75]. Apparently, individuals of *P. gastrophilus* are at their maximum reproductive capacity (see [76]), thus the higher allocation in egg size observed in worms from striped dolphins should involve higher costs for egg production. In support of this, the slope of the egg size-egg number regression, corrected for body size, seemed to be steeper in worms from striped dolphins, and indeed the best models examining the trade-off included the interaction between host species and egg area. However, this interaction was not statistically significant, which suggest that a great deal of unexplained variability also exist among individual hosts.

The previous discussion raises the question as to why adults of *P. gastrophilus* in the most suitable host opt to produce larger eggs rather than more numerous but smaller eggs. A possible explanation is that in an aquatic environment, the offspring can potentially be exposed to elevated levels of mortality (see, e.g. [19, 20, 77]). In particular, the first infective stage (which, depending on the putative life-cycle of *P. gastrophilus*, could be the egg, or the free-living miracidium that emerges from the egg) must face the challenge to contact the first intermediate host, and subsequent infective stages must make their way through the trophic web to infect a top predator. As noted above, Mediterranean striped and bottlenose dolphins favour different habitats, namely, oceanic vs. neritic [32, 78], which could pose different selective pressures. Oceanic ecosystems are widely recognised as having significantly lower levels of productivity than neritic ones [79], which also results in much lower density of organisms [64, 80]. From the perspective of *P. gastrophilus*, the oceanic habitat would therefore be much more adverse for transmission. The production of large eggs would therefore be an adaptive response because large eggs generate large, long-lived miracidia [35, 81], thus enhancing the likelihood of finding the first intermediate host (see [82]). In addition, the potential to multiply asexually within the first intermediate host could also be proportional to larval size [73], which would increase further transmission opportunities in the most adverse environment. Interestingly, a similar phenomenon has been reported in the case of the digenean *Proctoeces lintoni* Siddiqui and Cable, 1960: compared with pristine areas, parasite individuals from human-disturbed areas exhibited larger eggs because harvesting by humans had reduced the density of intermediate and definitive hosts [20]. Assuming that differences in the reproductive traits of *P. gastrophilus* in each dolphin species are adaptive, they could result from two potential processes, i.e. (i) phenotypic plasticity, so that the reproductive strategy of *P. gastrophilus* could vary according to the cues emanated from the definitive host (see [20]), or (ii) local adaptation by natural selection, so that differences in reproductive allocation between coastal vs. oceanic populations of *P. gastrophilus* could have at least a partial genetic component. The operation of these two processes should be explored in future studies.

## Conclusions

In summary, the results from the present study suggest that the reproductive strategy of *P. gastrophilus* could be differently optimised depending on the suitability of the host species and the local habitat where the life-cycle develops. As far as we are aware, this is the first study to document subtle differences in the reproductive strategy of a generalist helminth from marine mammals, illustrating how constraints and natural selection shape life history traits. Future research should explore whether differences between neritic and oceanic habitats have similar impact on the life-cycles of other trophically-transmitted helminths.
